# Variables related to health‐related quality of life among breast cancer survivors after participation in an interdisciplinary treatment combining mindfulness and physiotherapy

**DOI:** 10.1002/cam4.6035

**Published:** 2023-05-11

**Authors:** Josune Martín, Susana García, Ane Anton‐Ladislao, Josefa Ferreiro, Maximina Martín, Angel Padierna, José M. Quintana, the CAMISS‐prospective group

**Affiliations:** ^1^ Department of Neuroscience University of the Basque Country UPV/EHU Leioa Spain; ^2^ Research Unit Galdakao‐Usansolo Hospital Galdakao Bizkaia Spain; ^3^ Kronikgune Institute for Health Services Research Barakaldo Basque Country Spain; ^4^ Health Services Research on Chronic Diseases Network – REDISSEC Galdakao Bizkaia Spain; ^5^ Department of Oncology Galdakao‐Usansolo Hospital, Barrio Labeaga s/n Galdakao Bizkaia Spain; ^6^ Department of Gynaecology Galdakao‐Usansolo Hospital Galdakao Bizkaia Spain; ^7^ Department of Psychiatry Galdakao‐Usansolo Hospital Galdakao Bizkaia Spain

**Keywords:** breast cancer survivors, health‐related quality of life, interdisciplinary treatment, mindfulness, physiotherapy

## Abstract

**Background:**

Breast cancer diagnosis and treatment increase the potential psychological impact on breast cancer survivors (BCS). The objective of this study was to assess the effects of an interdisciplinary intervention during follow‐up in BCS and identify variables related to improvements in HRQoL.

**Materials and Methods:**

In a non‐randomised quasi‐experimental design performed on an outpatient basis in a hospital gynaecology and oncology unit, 60 BCS were assigned to an interdisciplinary experimental group (EG) or a usual care group (CG). The EG underwent 12 sessions of an interdisciplinary program which included Mindfulness and physiotherapy, for 120 min per day, once a week for 6 weeks. At baseline, at 6 weeks and at 3 months after the intervention, participants of EG and CG completed an assessment of HRQoL (EuroQol and EORTC‐QLQ‐C30) and symptomatology of anxiety and depression. Additionally, EG completed an assessment of satisfaction with the treatment. For data analysis, we used descriptive statistics, Wilcoxon test, Kruskal–Wallis test, Chi‐square and Fisher tests and generalised linear models.

**Results:**

After 6 weeks, statistically significant differences were apparent in global and cancer‐related health symptoms such as fatigue and pain in the EORTC QLQ‐C30 and in anxiety and depression, among the EG (*n* = 30) compared with the CG (*n* = 30). Patients receiving the intervention reported a high degree of satisfaction with the treatment. Three months after the intervention, patients in the EG continued to show statistically significant improvements compared with the CG. In addition, allocation to the EG was identified as a variable related to improvement of HRQoL (EORTC QLQ‐C30) in the multivariable model.

**Conclusions:**

The results of our study suggest that a 6‐week interdisciplinary intervention may improve HRQoL and symptomatology of anxiety and depression in BCS patients at 3 months. The study presents data that the intervention for BCS appears promising and warrants further study in a randomised controlled trial.

## 1 INTRODUCTION

Although breast cancer (BC) remains a highly prevalent form of cancer in women around the world, advances in early diagnosis and treatment have resulted in increased survival. In Spain, it remains the most frequent tumour among women and is one of the cancers with the highest rate of incidence, mortality and recurrence at 5 years.^1^

The majority of women diagnosed with early BC undergo treatment involving surgery and radiotherapy, chemotherapy and/or hormonal therapy, which is associated with a substantial burden of symptoms and impacts quality of life. Cancer survivors experience many adverse outcomes related to the disease or its treatment,^2^ as well as poorer health‐related quality of life (HRQoL).^3^ Having completed the principal treatments for BC, women have to cope with a stage of ‘watchful waiting’, a stressful period during which they may have the impression that the physician is ‘doing nothing’. Throughout this period, survivors continue to report physical symptoms and impaired QOL.^4^ The evidence suggests that the post‐treatment stage is an ideal moment for introducing stress‐reduction interventions.^5^ However, only a very limited number of treatment regimens have been developed to reduce this high level of morbidity during the difficult transitional period of post‐treatment survivorship.

A Danish population‐based cohort study of cancer survivors found that the need for psychological and physical rehabilitation was equally frequent.^6^ One *psychological intervention* among cancer patients comprises stress reduction using mindfulness (defined as deliberately paying attention to present‐time experiences in an accepting and non‐judgmental fashion).^7^ Recent meta‐analyses found that mindfulness‐based interventions are effective in reducing psychological distress among cancer patients,^8^ achieving decreases in anxiety and depressive symptoms and improvements in quality of life (QoL). *Physiotherapeutic interventions* are of particular relevance, since they influence both adverse physiologic and psychosocial outcomes, including HRQoL.^9^ There is increasing evidence that among patients with conditions such as breast, colon and prostate cancer and haematological malignancies, programs of physical exercise may offer benefits such as improvement in physical activity and HRQoL.^10^

Tremendous interest has been generated in the association between exercise and physiological and psychological well‐being in general and HRQoL in particular. As far as we know, there have been relatively few multidisciplinary interventions for cancer patients and survivors in Spain. In particular, no studies have investigated the effectiveness of a coordinated psychological and physiotherapeutic intervention.

In our opinion, given the high prevalence of BC and the negative impact on patient's HRQoL, methodologically rigorous interdisciplinary treatments should be developed and implemented that are specifically designed for breast cancer survivors (BCSs). We developed an interdisciplinary treatment for BCS based on the biopsychosocial model,^11^ combining coordinated psychological and physiotherapeutic components.

The chief objective of this study was to evaluate the effects of an interdisciplinary (psychological and physiological) group intervention for BCS compared with standard treatment. A further aim was to identify factors predicting improvement in HRQoL among these patients.

## 2 MATERIALS AND METHODS

### 2.1 Subjects

The study population was drawn prospectively from patients who had been referred to the gynaecology and oncology unit of Galdakao‐Usansolo Hospital. Women aged over 18 who had been diagnosed and treated for BC 2 years earlier were included. Patients who declined to participate in the study or were suffering from a severe psychiatric or organic disorder interfering with their ability to complete the questionnaires were not included. Patients were recruited between January 2016 and January 2017. The study received approval from the institutional review board of Galdakao‐Usansolo Hospital. All participants provided written informed consent. All information was kept confidential. This study forms part of the CaMISS‐study, an observational analytic prospective cohort study (Clinical Trials.govIdentifier:NCT02439554), and the study protocol conforms to the ethical guidelines of the 1975 Declaration of Helsinki (6th revision, 2008).

### 2.2 Study design and intervention

We used a non‐randomised quasi‐experimental design.^12^ While randomised trials are acknowledged to be the gold standard, some authors have raised concerns that they may not be the most suitable study design for evaluating psychosocial interventions in cancer patients; this is partly due to the fact that the patients' commitment to the intervention is considered to be an important predictor of benefit from the intervention.^13^ If patients are willing to be randomised to a control arm, it has been argued, they may not have as great a commitment to the intervention, which might result in an underestimation of its benefits.^13^

**FIGURE 1:**
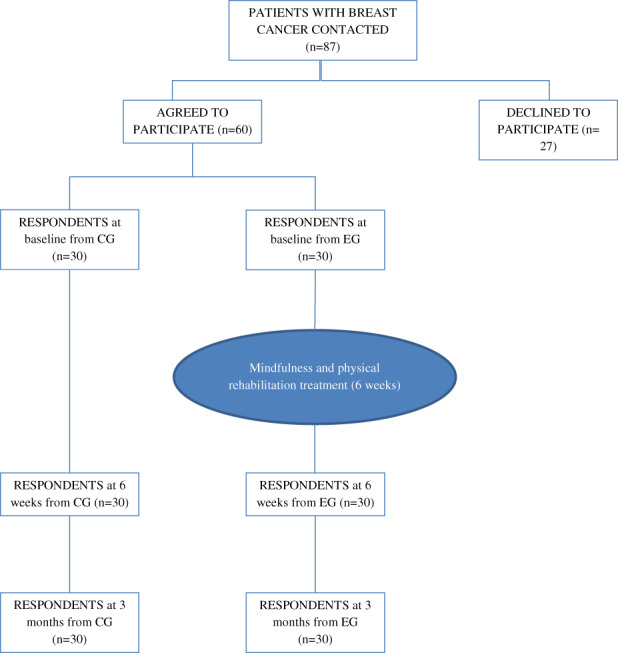
Flowchart of the sample of patients with breast cancer. CG, Control Group; EG, Experimental Group.

A total of 87 BCS were invited to participate in the study. Of these, 60 women who had survived BC voluntarily agreed to take part in the study (Figure 1). On determining the sample group, patients who agreed to come to the hospital to perform the interdisciplinary treatment were assigned to an experimental group (EG); those who did not agree (to come to the hospital to perform the interdisciplinary treatment) were allocated to a control group (CG). The patients from the CG received their usual care. Patients in the EG received the usual care and in addition, 12 concurrently sessions of psychological and physiological intervention over a period of 6 weeks. The team providing these sessions included a clinical psychologist and a physiotherapist with experience in managing BC. Outcome measures were assessed in all participants before, immediately after, and 3 months following the end of the group program.

### 2.3 The interdisciplinary group intervention

The *psychological component* focused on Mindfulness‐Based Stress Reduction (Breast Cancer) Intervention (MBSR, BC) and was carried out by an experienced clinical psychologist. The MBSR(BC) program, modelled on the MBSR program developed by Jon Kabat‐Zinn et al^14^ was adapted to assist BC patients to take an active role in stress reduction and symptom management through a self‐regulatory process of meditation.^15^ The intervention is made up of three components: (1) educational material related to relaxation, meditation, the mind–body connection and a healthy lifestyle, (2) meditation in group meetings and home assignments and (3) group processes related to barriers to the practice of meditation and supportive group interaction.^16^ A psychologist trained in mindfulness, taught the 6‐week, 75‐min‐per‐week sessions, which included training in formal meditation techniques, and informal techniques for integrating mindfulness into everyday activities. BCSs were requested to practise the meditative techniques and to record their practice times in a daily journal. A handbook was rovided to guide home practice.

In the *physiotherapeutic component*, which was based on research by Jones et al,^17^ patients carried out progressive physical training. This involved (non‐aerobic) warming, stretching and muscle‐strengthening exercises performed in a standing, sitting and reclining position, with no machine weights. A physiotherapist taught the 6‐week, 45‐min‐per‐week sessions, which included shoulder exercises combined with functional activities and educational strategy.

### 2.4 Instruments and data collection

#### 2.4.1 Clinical data

- Related to patients' personal background.
- Related to the care process.
- Related to neoadjuvant treatment.
- Related to anatomical pathology.
- Related to follow‐up at 2 years and adjuvant treatment.

#### 2.4.2 Patient‐reported measures

- The self‐report version of the *EuroQol generic health‐related quality of life questionnaire (EQ‐5D)* ^18^ comes in two parts: the EQ‐5D‐5L descriptive system (Scale I) and the EQ Visual Analogue scale (VAS) (Scale II). The descriptive system consists of five dimensions. In each one, the patient chooses one of five answers, indicating varying degrees of severity. The EQ VAS maps the respondent's self‐rated health on a 20‐cm vertical, visual analogue scale. The scale ranges from 0 (worst imaginable state of health) to 100 (best imaginable state of health).
- *The EORTC‐QLQ‐C30 (Version 3.0)* ^19^, ^20^ is an internationally validated HRQoL questionnaire which is widely used in cancer research. The core questionnaire comprises 30 items that assess five functioning domains; eight cancer symptom domains; financial difficulties and global QoL. The scores are converted into a 0–100 scale, in which a high score indicates a high level of functioning or global QoL, whereas in the symptom dmains, higher scores indicate a greater burden of symptoms.
- *The Hospital Anxiety and Depression Scale (HADS)* is a 14‐item instrument used to screen for anxiety and depression in non‐psychiatric settings.^21^ It is divided into 2 subscales. A score of 0 to 7 on any subscale indicates an absence of symptoms; a score of 8 to 10 suggests a possible case and a score of 11 or more indicates the presence of symptoms.^22^
- To determine the patients' opinions of the intervention they had received, an ad‐hoc *satisfaction scale* was created, comprising a single question: ‘Are you satisfied with the treatment?’. Participants were asked to reply on a 5‐point Likert scale, from *not satisfied* to *entirely satisfied*.

### 2.5 Statistical analysis

Descriptive statistics included frequencies and percentages for categorical data and means and standard deviations for continuous variables. Differences between experimental and control groups were evaluated using the Chi‐square or Fisher exact test for categorical variables and Student *t*‐test or non‐parametric Wilcoxon test for continuous variables.

In all the questionnaires, the differences between baseline and 6 weeks and baseline and 3 months after intervention were calculated; the Wilcoxon signed‐rank test was used. Differences between the groups were evaluated by means of the Student *t*‐test or non‐parametric Wilcoxon test. The effect size (ES) was calculated between baseline scores and scores at 6 weeks and 3 months. EFs were expressed as Cohen's d.^23^

After a univariable analysis to identify factors related to the change in global health status measured by the EORTC‐QLQ30 between the baseline score and the score at 6 weeks and 3 months, a multivariable generalised lineal model was performed. Group of patients, global health status at baseline measured by EORTC‐QLQ‐C30, social functioning at baseline measured by EORTC‐QLQ‐C30 and depression at baseline measured by HADS, were the factors included in the final multivariable generalised linear model. All those variables with a *p* < 0.20 in univariable analysis were selected for multivariable model and in the multivariable final model were included those with a *p* < 0.05 after a backward selection. R‐squared was used to determine the percentage of explained variance by the model.

All effects were considered statistically significant at *p* < 0.05. All statistical analyses were performed using SAS System, version 9.4 software (Copyright, SAS Institute Inc.). The figure was created using R3.4.0.

## 3 RESULTS

The study sample comprised 87 patients with BC. Of these, 60 patients agreed to participate; all completed the instruments at baseline and 6 weeks and 3 months following the intervention (Figure 1).

Table 1 shows the *sociodemographic variables and clinical histories* of the BC patients participating in the study. Mean age was 54.55 (SD = 9.96). The majority of participants had been diagnosed with Stage I (51.67%) of invasive ductal carcinoma (55.93%) approximately 2 years prior to the current study. 91.53% received adjuvant postoperative treatment and at least 75% had received no psycho‐oncology or rehabilitation sessions. No statistically significant differences were observed in the baseline (sociodemographic and clinical) characteristics of the EG and the CG (Table 1). However, statistically significant differences were seen in the baseline symptomatology for anxiety and depression (Table S1).

**TABLE 1:** Baseline data on patients with breast cancer, by group (experimental or control).

	Total Patients (*n* = 60)	Experimental group (*n* = 30)	Control group (*n* = 30)	*p*‐value
	*n* (%)	*n* (%)	*n* (%)	
*Sociodemographic data*				
Age (years) $\bar{x}$(SD)	54.55 (9.96)	53.43 (8.99)	55.66 (10.89)	0.23
Marital status				0.60
Single	1 (1.67)	1 (3.33)	0 (0)	
Married	49 (81.67)	23 (76.67)	26 (86.66)	
Separated or divorced	6 (10.00)	4 (13.33)	2 (6.67)	
Widow/widower	4 (6.67)	2 (6.67)	2 (6.67)	
In employment (yes)	47 (78.33)	24 (80.00)	23 (76.67)	0.75
Children (yes)				
Number of children				0.09
1	15 (27.27)	8 (29.63)	7 (25.00)	
2	34 (61.82)	19 (70.37)	15 (53.57)	
3	5 (9.09)	0 (0)	5 (17.86)	
4	1 (1.82)	0 (0)	1 (3.57)	
*Clinical data*				
Smoker				0.82
Non‐smoker	12 (32.43)	5 (27.78)	7 (36.84)	
Current smoker	13 (35.14)	7 (38.89)	6 (31.58)	
Former smoker	12 (32.43)	6 (33.33)	6 (31.58)	
Family history of gynaecological cancer (yes)	14 (23.33)	7 (23.33)	7 (23.33)	1.00
Charlson Index categorised				0.10
0	50 (83.33)	28 (93.33)	22 (73.33)	
1	9 (15.00)	2 (6.67)	7 (23.33)	
2	0 (0)	0 (0)	0 (0)	
>2	1 (1.67)	0 (0)	1 (3.33)	
*Pathological anatomy data*				
Laterality				0.11
Left breast	36 (60.00)	21 (70.00)	15 (50.00)	
Right breast	24 (40.00)	9 (30.00)	15 (50.00)	
Bilateral	0 (0)	0 (0)	0 (0)	
Cancer stage				0.30
0	12 (20.00)	7 (23.33)	5 (16.67)	
I	31 (51.67)	15 (50.00)	16 (53.33)	
II	14 (23.33)	8 (26.67)	6 (20.00)	
III	3 (5.00)	0 (0)	3 (10.00)	
IV	0 (0)	0 (0)	0 (0)	
Histological types				0.23
Ductal carcinoma in situ	12 (20.34)	9 (30.00)	3 (10.34)	
Invasive ductal carcinoma	33 (55.93)	15 (50.00)	18 (62.07)	
Invasive lobular carcinoma	6 (10.17)	4 (13.33)	2 (6.90)	
Tubular carcinoma	2 (3.39)	0 (0)	2 (6.90)	
Mucinous carcinoma	1 (1.69)	1 (3.33)	0 (0)	
Medullary	1 (1.69)	0 (0)	1 (3.45)	
Invasive cribriform	0 (0)	0 (0)	0 (0)	
Invasive papillary	1 (1.69)	0 (0)	1 (3.45)	
Carcinoma not specified	0 (0)	0 (0)	0 (0)	
Other	3 (5.08)	1 (3.33)	2 (6.90)	
*Neoadjuvant therapy data*				
Pre‐intervention treatment type (yes)	2 (3.33)	1 (3.33)	1 (3.33)	1.00
Chemotherapy (yes)	2 (3.33)	1 (3.33)	1 (3.33)	1.00
Radiation (yes)	1 (1.67)	1 (3.33)	0 (0)	1.00
Hormone therapy (yes)	0 (0)	0 (0)	0 (0)	na
*Follow‐up at 2 years data*				
Adjuvant postoperative treatment (yes)	54 (91.53)	28 (93.33)	26 (89.66)	0.67
Radiation (yes)	43 (81.13)	21 (77.78)	22 (84.62)	0.73
Chemotherapy (yes)	24 (44.44)	13 (46.43)	11 (42.31)	0.76
Hormone therapy (yes)	48 (88.89)	24 (85.71)	24 (92.31)	0.67
Tamoxifen (yes)	25 (52.08)	11 (45.83)	14 (58.33)	0.39
Analog + tamoxifen (yes)	3 (6.25)	1 (4.17)	2 (8.33)	1.00
Tamoxifen + aromatase inhibitors (yes)	0 (0)	0 (0)	0 (0)	na
Inhibitors (yes)	26 (54.17)	14 (58.33)	12 (50.00)	0.56
Others	2 (4.17)	1 (4.17)	1 (4.17)	1.00
Anti‐HER2 (yes)	6 (11.11)	1 (3.57)	5 (19.53)	0.09
Herceptin (yes)	6 (100)	1 (100)	5 (100)	na
Lapatinib (yes)	0 (0)	0 (0)	0 (0)	na
Pertuzumab (yes)	0 (0)	0 (0)	0 (0)	na
Others (yes)	0 (0)	0 (0)	0 (0)	na
Other postoperative treatments (yes)	10 (16.67)	6 (20.00)	4 (13.33)	0.49
Rehabilitation (yes)	7 (70.00)	3 (50.00)	4 (100)	0.20
Psychologist/psychiatrist (yes)	4 (40.00)	3 (50.00)	1 (25.00)	0.57
Tumour recurrence (yes)	1 (1.69)	1 (3.33)	0 (0)	1.00
Treatment for recurrence (yes)	1 (100)	1 (100)	0 (0)	na
Number of rehabilitation sessions				
First year at follow‐up	0.53 (2.46)/ 0 [0–0]*	0.57 (2.57)/ 0 [0–0]*	0.50 (2.39)/ 0 [0–0]	0.66
Second year at follow‐up	0.85 (3.93)/ 0 [0–0]*	0.23 (1.28)/ 0 [0–0]*	1.47 (5.38)/ 0 [0–0]	0.17
Number of sessions with psycho‐oncologist				
First year at follow‐up	0.20 (1.10)/0 [0–8]^¥^	0.37 (1.54)/0 [0–8]^¥^	0.03 (0.18)/0 [0–1]^¥^	0.54
Second year at follow‐up	0.10 (0.66)/0 [0–5]^¥^	0.20 (0.92)/0 [0–5]^¥^	0 (0)/0 [0–0]^¥^	0.16
Time since diagnosis (years)	2.22 (0.31)/2.21 [1.95–2.48]*	2.28 (0.29)/2.23 [2.09–2.48]*	2.16 (0.33)/2.15 [1.90–2.35]*	0.14

Abbreviations: $\bar{x}$, mean; HER2, human epidermal growth factor receptor‐2; SD, standard deviation.

* Data are given as median [interquartile range]. Level of significance *p* < 0.05.

^¥^ Data are given as median [minimum‐maximum].

Six weeks after intervention, a statistical improvement was observed in the EG in general HRQoL(*p* ≤ 0.0001) and specific HRQoL for cancer(*p* ≤ 0.0001) and in anxiety (*p* = 0.001), compared with the CG. In addition to these improvements, 3 months after the intervention, a statistical improvement was observed in emotional functioning (*p* ≤ 0.0001), dyspnoea (*p* = 0.03) and symptomatology of anxiety and depression (*p* ≤ 0.0001 in both) (Table S1).

Table 2 represents *change in HRQoL* and anxiety and depression between baseline and 6 weeks and between baseline and 3 months among patients receiving and not receiving the intervention. ES of change in the EG were above 0.5 in the EQ‐5D of the EuroQol‐5D‐5L; the functional scales (role functioning, emotional functioning and cognitive functioning); and the symptom scales (fatigue and insomnia) of the EORTC QLQ‐C30. ES were higher than 0.8 in the VAS of the EuroQol‐5D‐5L, in the global health status of the EORTC QLQ‐C30, and in both scales of the HADS. Three months after the intervention, patients in the EG continued to report statistically significant changes in global health status (Figure S1).

**TABLE 2:** Change and effect size of the questionnaires between baseline and 6 weeks and 3 months, between experimental and control groups.

	Change Baseline‐6 weeks		Change Baseline‐6 weeks		*p*‐value^+^	Change Baseline‐3 months		Change Baseline‐3 months		*p*‐value^+^	Effect size Baseline‐6 weeks		Effect size Baseline‐3 months	
	EG (*n* = 30)		CG (*n* = 30)			EG (*n* = 30)		CG (*n* = 30)			EG (*n* = 30)	CG (*n* = 30)	EG (*n* = 30)	CG (*n* = 30)
	$\bar{x}\;\left(\mathrm{SD}\right)$	*p*‐value*	$\bar{x}\;\left(\mathrm{SD}\right)$	*p*‐value*		$\bar{x}\;\left(\mathrm{SD}\right)$	*p*‐value*	$\bar{x}\;\left(\mathrm{SD}\right)$	*p*‐value*		ES	ES	ES	ES
Euroqol‐5D‐5L														
Scale 1 (EQ‐5D)	0.09 (0.11)	**<0.0001**	−0.002 (0.03)	0.84	**<0.0001**	0.13 (0.14)	**<0.0001**	−0.03 (0.05)	**<0.0001**	**<0.0001**	−0.56	0.05	−0.72	0.19
Scale 2 (VAS)	16.17 (14.95)	**<0.0001**	−2.50 (7.96)	0.07	**<0.0001**	20.17 (15.34)	**<0.0001**	−5.67 (9.71)	**0.008**	**<0.0001**	−0.92	0.10	−1.15	0.23
EORTC QLQ‐C30														
Global health status	23.33 (17.97)	**<0.0001**	−3.06 (5.57)	**0.007**	**<0.0001**	30.28 (15.55)	**<0.0001**	−6.11 (9.01)	**0.0005**	**<0.0001**	−1.45	0.12	−1.88	0.25
Functional scales														
Physical functioning	6.00 (10.26)	**0.0004**	−0.44 (1.69)	0.50	**0.0005**	4.67 (11.63)	**0.02**	−0.89 (2.30)	0.12	**0.0005**	−0.35	0.03	−0.27	0.05
Role functioning	10.56 (17.77)	**0.002**	−1.11 (4.23)	0.50	**0.002**	13.33 (19.27)	**0.0007**	−1.11 (4.23)	0.50	**0.002**	−0.41	0.05	−0.52	0.05
Emotional functioning	18.33 (25.28)	**0.0002**	−5.83 (5.43)	**<0.0001**	**<0.0001**	19.17 (28.63)	**0.0004**	−17.22 (11.97)	**<0.0001**	**<0.0001**	−0.74	0.24	−0.77	0.71
Cognitive functioning	12.78 (18.92)	**0.0008**	−1.67 (8.01)	0.50	**0.001**	16.67 (20.99)	**<0.0001**	−4.44 (10.66)	**0.02**	**0.001**	−0.55	0.08	−0.71	0.20
Social functioning	10.56 (18.82)	**0.003**	0 (4.38)	1.00	**0.003**	12.22 (18.54)	**0.0002**	1.11 (9.72)	1.00	**0.003**	−0.38	0	−0.45	−0.05
Symptom scales														
Fatigue	−13.70 (19.94)	**0.0006**	1.85 (5.12)	0.12	**0.0004**	−15.56 (21.76)	**<0.0001**	4.07 (6.18)	**0.002**	**0.0004**	0.56	−0.08	0.64	−0.17
Nausea and vomiting	−2.78 (14.57)	0.50	0 (0)	na	0.16	−3.89 (12.90)	0.12	−0.56 (3.05)	1.00	0.16	0.22	0	0.30	0.08
Pain	−10.00 (26.48)	0.06	1.11 (4.23)	0.50	**0.03**	−11.67 (20.60)	**0.004**	3.33 (6.78)	**0.03**	**0.03**	0.35	−0.04	0.41	−0.13
Dyspnoea	−12.22 (23.95)	**0.01**	0 (0)	na	**0.006**	−12.22 (26.96)	**0.02**	6.90 (13.74)	**0.03**	**0.006**	0.43	0	0.43	−0.28
Insomnia	−20.00 (25.67)	**0.0002**	0 (8.75)	1.00	**0.0002**	−25.56 (29.92)	**<0.0001**	1.11 (10.66)	1.00	**0.0002**	0.60	0	0.76	−0.05
Appetite loss	−8.89 (19.44)	0.03	0 (0)	na	**0.01**	−11.11 (20.22)	**0.007**	0 (0)	na	**0.01**	0.34	0	0.43	0
Constipation	−10.00 (23.41)	**0.006**	−1.11 (6.09)	1.00	0.05	−10.00 (23.41)	**0.04**	−3.33 (10.17)	0.25	0.05	0.34	0.05	0.34	0.15
Diarrhoea	−2.22 (12.17)	0.62	1.11 (6.09)	1.00	0.18	−4.44 (11.52)	0.12	0 (0)	na	0.18	0.19	−0.08	0.39	0
Financial difficulties	−4.44 (19.04)	0.37	1.11 (6.09)	1.00	0.18	−2.22 (14.99)	0.75	−1.11 (6.09)	1.00	0.18	0.12	−0.05	0.06	0.05
HADs														
Symptomatology of anxiety	−7.33 (4.81)	**<0.0001**	0.93 (1.05)	**<0.0001**	**<0.0001**	−7.83 (4.51)	**<0.0001**	3.23 (1.91)	**<0.0001**	**<0.0001**	1.72	−0.20	1.84	−0.70
Symptomatology of depression	−6.67 (4.85)	**<0.0001**	0.53 (1.07)	**0.004**	**<0.0001**	−7.03 (4.47)	**<0.0001**	2.57 (1.30)	**<0.0001**	**<0.0001**	1.35	−0.15	1.42	−0.73

*Note*: *p*‐values in bold indicate a significance level of *p* < 0.05.

Abbreviations: $\bar{x}$, mean; CG, Control Group; EG, Experimental Group; EORTC QLQ‐C30, European Organisation for Research and Treatment‐a cancer‐specific measure of Health‐related Quality of Life Questionnaire; ES, effect size (0.20 = small effect; 0.50 = medium effect; *d* ≥ 0.8 = large effect); Euroqol‐5D‐5L, EuroQoL 5‐domain for health‐related quality of life; HADS, Hospital Anxiety and Depression Scale; SD, standard deviation; VAS: visual analogue scale.

* *p*‐value corresponding to intragroups differences.

^+^ *p*‐value corresponding to differences between groups.

The results of the *univariable analysis* of Global Health Status for the EG and CG at 6 weeks and 3 months, by baseline sociodemographic variables are shown in Table S2.

The results of the *multivariable analysis* of the entire sample are shown in Table 3 (adjusted for sociodemographic and baseline clinical factors). There was a significant association between belonging to the EG and changes in the HRQoL the between baseline, 6 weeks, and 3 months after the intervention. Global health status, social functioning and symptomatology of depresson were significant variables related to a reduction in the HRQoL, so that those patients in the EG who had more depression and lower QoL at baseline time, experienced an improvement in HRQoL.

**TABLE 3:** Multivariable model of health‐related quality of life at 6 weeks and 3 months after treatment.

	Global health status (EORTC QLQ‐C30)					
	6 weeks after treatment			3 months after treatment		
	*β* (SE)	95% CI	*p*‐value	*β* (SE)	95% CI	*p*‐value
Intercept	3.72 (6.55)		0.57	16.50 (4.14)		**0.002**
Global health status baseline (EORTC QLQ‐C30)	0.68 (0.07)	(0.54, 0.83)	**<0.0001**	0.62 (0.06)	(0.50, 0.74)	**<0.0001**
Social functioning baseline (EORTC QLQ‐C30)	0.13 (0.06)	(0.004, 0.25)	**0.04**	**—**		
Depression baseline (HADS)						
8–10 vs. ≤7	9.53 (4.22)	(1.07, 18.00)	**0.02**	4.36 (3.42)	(−2.49, 11.21)	0.20
≥11 vs. ≤7	11.55 (4.07)	(3.39, 19.72)	**0.006**	7.10 (3.45)	(0.18, 14.02)	**0.04**
Group (experimental vs. control)	21.05 (3.17)	(14.70, 27.41)	**<0.0001**	31.58 (2.71)	(26.16, 37.00)	**<0.0001**
*R* ^2^		0.76			0.84	

*Note*: Baseline refers to the baseline measurement of the corresponding variable. p‐values in bold indicate a significance level of *p* < 0.05.

Abbreviations: —, not applicable; *β* (SE): Beta (standard error); 95% CI: 95% confidence interval; EORTC QLQ‐C30: European Organisation for Research and Treatment: a cancer specific measure of Health‐related Quality of Life Questionnaire; HADS: Hospital Anxiety and Depression scale; *R* ^2^: Explained variance.

Moreover, patients who received the interdisciplinary intervention reported being *very satisfied* with their treatment. In our study, patients reported that they were satisfied with the intervention. 70% of the EG patients were ‘entirely satisfied’ with treatment at 3 months, and 30% were ‘very satisfied’ (Table S3).

## 4 DISCUSSION

To summarise, BC and its treatment pose many challenges to the patient's physical, emotional, mental and social well‐being and negatively impact the patient's QoL. Furthermore, HRQoL and its domains are important measures for cancer survivorship as they provide prognostic^24^ and predictive^25^ information, together with the survivors' subjective experiences^26^ of therapeutic and lifestyle interventions.

An interdisciplinary treatment that included coordinated psychological and physiotherapeutic interventions improved HRQoL, anxiety and depression among BCS 6 weeks and 3 months after the interdisciplinary treatment, when compared to the usual treatment for such patients. To our knowledge, this is the first trial conducted in a group setting in a hospital environment in the Basque Country that assesses the efficacy of an interdisciplinary treatment for BCS. This study investigated changes in HRQoL, anxiety and depression after a 6‐week interdisciplinary intervention in 60 BCSs. Additionally, the baseline scores of HRQoL, depression and social functioning and the study group were significant variables related to improvement of HRQoL.

Even following the completion of treatment for BC, survivors regularly suffer severe psychological stress, anxiety, depression, fear of recurrence, physical pain, fatigue and reduced QoL.^27^ There is relevant research in the during‐ as well as after treatment period regarding interventions that can help modulate symptoms and avoid QOL deterioration after BC, including, for example, interventions for patients on endocrine therapy^28^ or for specific symptoms such as cancer‐related fatigue.^29^ Most of these interventions are behavioural interventions that include psychosocial support and exercise interventions. Some of these were multicomponent and interdisciplinary,^30^ or it has concentrated on pain relief and improvements in physical and psychological functions during BC treatment.^31^

Our results are consistent with those of previous studies,^32^ which indicated that multicomponent therapy is effective in reducing anxiety and depression and for improving HRQoL after the end of treatment. Nonetheless, our study cannot readily be compared with others, generally due to the heterogeneous nature of the treatment, instruments of measurement and study designs employed, given that no international standards exist for interdisciplinary programs.^33^

Although all patients in our study had early BC, the emotional response to BC is not dependent on the stage of the disease, given that women who have been diagnosed with non‐invasive BCs also experience powerful emotions.^34^, ^35^ Previous studies have shown that BCS are liable to encounter mental health problems, especially *depressive and anxious symptoms*, which are by no means abnormal responses to the intensive surgical and medical treatment, uncertainty and loss of control.^36^ Distress is experienced by patients with cancer across diagnoses and across the trajectory of the disease,^37^ reducing the BCS' sense of well‐being and QoL. Classen et al^38^ indicate that 22%–50% of patients with BC fulfil the criteria for a psychiatric diagnosis of depression. However, in the present study, the results for patients 2 years after their diagnosis of BC have shown that there is a group of women with a high level of anxiety (mean = 11.93, SD = 4.26) and a moderate level of depressive symptoms (mean = 8.93, SD = 4.94) who could benefit from psycho‐oncologic interventions aimed at improving this symptomatology.

Three months after completion of the interdisciplinary intervention, patients continued to show statistically significant improvements in *HRQoL*, anxiety and depression.

With regard to *changes of QoL* at 3 months, the interdisciplinary treatment improved HRQoL, functional scales, certain symptoms, anxiety and depression. This matches the findings of other studies on multimodal treatment that also assess HRQoL using the EORTC‐QLQ‐C30.^39^ The results showed significantly lower anxiety and depression rates among patients from the EG than those in the CG. This is consistent with a study previously undertaken on 24 BC patients by Jang et al.^40^ Our findings are also similar to those of a study by Lengacher et al,^15^ which showed that MBSR reduced patients' levels of depression, anxiety and fear of recurrence and improved their levels of energy and physical function.

When interpreting the results in terms of ES, the ES for HRQoL, anxiety and depression were large (*d* = 1.88, *d* = 1.84, *d* = 1.42, respectively). Matchim et al^41^ made a systematic review of MBSR for BCS, reporting studies with large ES on anxiety and moderate ES on mood symptoms.

With regard to variables *predicting* HRQoL improvement in BCS patients, the findings of this study suggest that HRQoL, depression, social functioning, and especially, belonging to the EG, might be relevant to HRQoL. These results suggest that being in an EG is the main variable related to improvement in HRQoL. The variable that best predicted an improvement in the HRQoL 3 months after the intervention was the study group.

It is important to stress that the EG patients in our study reported being *satisfied* with the interdisciplinary treatment. This issue is an important one in healthcare, since satisfied patients show a greater likelihood of co‐operating with their doctors and participating in their own treatment.^42^, ^43^ Information on patient satisfaction is seen as a way of including patients' perspectives in the planning and assessment of services.^43^

Our findings support the preliminary evidence that interdisciplinary treatment can be significantly efficacious in alleviating anxiety and depression and improving HRQoL in women survivors of BC. Moreover, the ES is large. Our purpose was to furnish patients with the skills that would assist their long‐term adaptation to BC and improve their HRQoL.

One limitation of this research is that only a small number of BCSs were included. Because the women were not allocated randomly to the program, it is difficult to draw conclusions drawn from the results of the study. Although a good balance between the groups was maintained with regard to disease, treatment and demographic variables, patients with anxiety (66%) were more likely to accept an interdisciplinary intervention; that is, there is a group of patients who have been diagnosed and treated for BC, who have shown elevated levels of symptomatology of anxiety and depression within 2 years (approximately) of their cancer diagnosis, which prevents a valid comparison between the two groups. Although this study was not undertaken as RCT, which is a very substantial limitation, the significant ESs that we observe in the EG are exciting and the intervention does warrant further study in a randomised controlled trial. A limitation of our study was that there was no included the molecular subtypes of BC. Another limitation is that the lack of standard interdisciplinary treatments for BCSs and standard study designs hinders comparison between our study and others. Another limitation is that the majority of the sample (72%) were stage 0 and 1. A limitation is that there was no assessment of satisfaction in the standard care group. Another limitation was that few patients received anti HER2 treatment in the EG. Finally, future longitudinal research in a large sample is needed for BCSs and to observe ways in which the interdisciplinary treatment can be applied clinically.

## AUTHOR CONTRIBUTIONS

**Josune Martín:** Conceptualization (lead); data curation (supporting); formal analysis (supporting); funding acquisition (supporting); investigation (lead); methodology (equal); project administration (equal); resources (supporting); software (supporting); supervision (lead); validation (lead); visualization (equal); writing – original draft (lead); writing – review and editing (lead). **Susana García:** Conceptualization (supporting); data curation (supporting); formal analysis (supporting); funding acquisition (lead); investigation (supporting); methodology (lead); project administration (lead); resources (lead); software (supporting); supervision (supporting); validation (supporting); visualization (equal); writing – original draft (supporting); writing – review and editing (equal). **Ane Anton‐Ladislao:** Conceptualization (supporting); data curation (lead); formal analysis (lead); funding acquisition (supporting); investigation (supporting); methodology (supporting); project administration (supporting); resources (supporting); software (lead); supervision (supporting); validation (supporting); visualization (supporting); writing – original draft (supporting); writing – review and editing (equal). **Josefa Ferreiro:** Conceptualization (supporting); data curation (supporting); formal analysis (supporting); funding acquisition (supporting); investigation (supporting); methodology (supporting); project administration (supporting); resources (supporting); software (supporting); supervision (supporting); validation (supporting); visualization (supporting); writing – original draft (supporting); writing – review and editing (equal). **Maximina Martín:** Conceptualization (supporting); data curation (supporting); formal analysis (supporting); funding acquisition (supporting); investigation (supporting); methodology (supporting); project administration (supporting); resources (supporting); software (supporting); supervision (supporting); validation (supporting); visualization (supporting); writing – original draft (supporting); writing – review and editing (equal). **Angel Padierna:** Conceptualization (supporting); data curation (supporting); formal analysis (supporting); funding acquisition (supporting); investigation (supporting); methodology (supporting); project administration (supporting); resources (supporting); software (supporting); supervision (supporting); validation (supporting); visualization (lead); writing – original draft (supporting); writing – review and editing (equal). **José M. Quintana:** Conceptualization (supporting); data curation (supporting); formal analysis (supporting); funding acquisition (supporting); investigation (supporting); methodology (supporting); resources (supporting); software (supporting); supervision (supporting); validation (supporting); visualization (supporting); writing – original draft (equal).

## FUNDING INFORMATION

This study was partly financed by funds awarded by the Asociación Española Contra el Cáncer (Spanish Association Against Cancer, Junta de Bizkaia) (AECC14/901) and the Carlos III Health Institute (PI12/01842) to principal investigator Susana García, and the University of the Basque Country UPV/EHU.

## CONFLICT OF INTEREST STATEMENT

The authors declare that they have no competing interests.

## ETHICAL APPROVAL STATEMENT

All procedures performed in studies involving human participants were in accordance with the ethical standards of the institutional and/or national research committee and with the 1964 Helsinki declaration, subsequent amendments thereto and comparable ethical standards.

## CLINICAL TRIAL REGISTRATION NUMBER

NCT02439554.

## Supporting information


Figure S1

Table S1

Table S2

Table S3

## Data Availability

The data that support the findings of this study are available from the corresponding author at email direction (jmartin@cop.es).
