# Supplementary figures and images for: Variables related to health‐related quality of life among breast cancer survivors after participation in an interdisciplinary treatment combining mindfulness and physiotherapy
Source: Cancer Med. 2023 May 11;12(12):13834–45. doi: 10.1002/cam4.6035 (PMC10315809; doi:10.1002/cam4.6035)

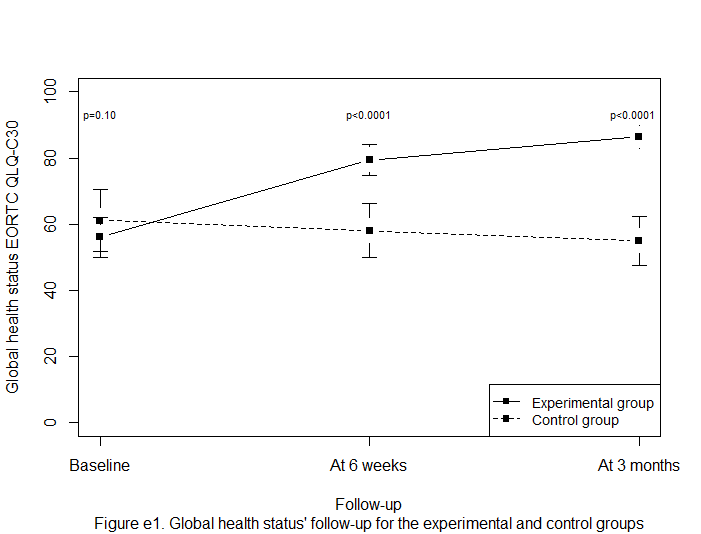

Supplement: Supplementary file 1 — Figure S1 [file CAM4-12-13834-s002.tiff]
