# Supplementary material for: Variables related to health‐related quality of life among breast cancer survivors after participation in an interdisciplinary treatment combining mindfulness and physiotherapy
Source: Cancer Med. 2023 May 11;12(12):13834–45. doi: 10.1002/cam4.6035 (PMC10315809; doi:10.1002/cam4.6035)
Supplement: Supplementary file 2 — Table S1 [file CAM4-12-13834-s004.docx]

**e-Table 1. Comparison between experimental and control group scores in health-related quality of life, symptomatology of anxiety and depression, at baseline, at 6 weeks and at 3 months.**

|  | **Baseline (prior to Mindfulness intervention)** | | | **6 weeks after Mindfulness intervention** | | | **3 months after Mindfulness intervention** | | |
| --- | --- | --- | --- | --- | --- | --- | --- | --- | --- |
|  | **EG** (n = 30) | **CG** (n = 30) | **p- value** | **EG** (n = 30) | **CG** (n = 30) | **p- value** | **EG** (n = 30) | **CG** (n = 30) | **p- value** |
|  | $\bar{x}$ **(SD)** | $\bar{x}$ **(SD)** |  | $\bar{x}$ **(SD)** | $\bar{x}$ **(SD)** |  |  |  |  |
| **Euroqol-5D-5L** |  |  |  |  |  |  |  |  |  |
| Scale 1 (EQ-5D) | 0.77 (0.18) | 0.87 (0.21) | **0.005** | 0.87 (0.15) | 0.86 (0.22) | 0.53 | 0.90 (0.13) | 0.83 (0.22) | 0.08 |
| Scale 2 (VAS) | 63.33 (17.53) | 63.17 (25.17) | 0.53 | 79.51 (16.10) | 60.67 (18.88) | **<0.0001** | 83.50 (12.74) | 57.50 (16.70) | **<0.0001** |
| **EORTC QLQ-C30** |  |  |  |  |  |  |  |  |  |
| **Global health status** | 56.11 (16.07) | 61.11 (24.79) | 0.10 | 79.44 (12.90) | 58.06 (22.05) | **<0.0001** | 86.39 (9.15) | 55.00 (19.65) | **<0.0001** |
| **Functional scales** |  |  |  |  |  |  |  |  |  |
| Physical functioning | 84.67 (17.08) | 88.44 (17.50) | 0.15 | 90.67 (12.58) | 88.00 (17.97) | 1.00 | 89.33 (14.07) | 87.56 (18.17) | 0.92 |
| Role functioning | 78.89 (25.50) | 90.00 (22.57) | 0.03 | 89.44 (14.83) | 88.89 (23.71) | 0.50 | 92.22 (13.66) | 88.89 (23.71) | 0.93 |
| Emotional functioning | 66.94 (24.81) | 79.72 (24.14) | 0.03 | 85.28 (15.27) | 73.89 (24.24) | 0.05 | 86.11 (18.74) | 62.50 (22.82) | **<0.0001** |
| Cognitive functioning | 76.11 (23.44) | 87.77 (21.86) | 0.01 | 88.89 (11.85) | 86.11 (23.19) | 0.68 | 92.78 (12.13) | 83.33 (23.16) | 0.06 |
| Social functioning | 77.78 (27.45) | 86.67 (23.33) | 0.12 | 88.33 (17.59) | 86.67 (22.49) | 0.95 | 90.00 (16.72) | 87.78 (22.29) | 0.82 |
| **Symptom scales** |  |  |  |  |  |  |  |  |  |
| Fatigue | 31.48 (24.43) | 19.63 (24.53) | 0.02 | 17.78 (12.92) | 21.48 (24.58) | 0.95 | 15.93 (19.72) | 23.70 (25.22) | 0.23 |
| Nausea and vomiting | 3.89 (12.90) | 2.22 (7.24) | 0.70 | 1.11 (6.09) | 2.22 (7.24) | 0.32 | 0 (0) | 1.67 (6.71) | 0.16 |
| Pain | 27.22 (28.19) | 16.11 (25.70) | 0.04 | 17.22 (18.30) | 17.22 (26.44) | 0.45 | 15.56 (17.47) | 19.44 (25.92) | 0.90 |
| Dyspnoea | 20.00 (28.50) | 12.22 (22.29) | 0.24 | 7.78 (14.34) | 12.22 (23.95) | 0.67 | 7.78 (20.87) | 18.39 (26.10) | **0.03** |
| Insomnia | 46.67 (33.45) | 20.00 (27.12) | **0.001** | 26.67 (23.81) | 20.00 (28.50) | 0.17 | 21.11 (26.96) | 21.11 (29.66) | 0.83 |
| Appetite loss | 14.44 (25.80) | 3.33 (10.17) | 0.05 | 5.56 (12.63) | 3.33 (10.17) | 0.45 | 3.33 (13.42) | 3.33 (10.17) | 0.68 |
| Constipation | 20.00 (29.81) | 12.22 (22.29) | 0.32 | 10.00 (17.83) | 11.11 (22.03) | 1.00 | 10.00 (21.71) | 8.89 (21.32) | 0.77 |
| Diarrhoea | 4.44 (11.52) | 3.33 (13.42) | 0.43 | 2.22 (8.46) | 4.44 (14.47) | 0.63 | 0 (0) | 3.33 (13.42) | 0.16 |
| Financial difficulties | 15.56 (35.81) | 11.11 (23.71) | 0.74 | 11.11 (28.14) | 12.22 (25.50) | 0.59 | 13.33 (32.28) | 10.00 (23.41) | 0.88 |
| **HADs** |  |  |  |  |  |  |  |  |  |
| Symptomatology of anxiety | 11.93 (4.26) | 6.90 (4.63) | **<0.0001** | 4.60 (3.18) | 7.83 (4.23) | **0.001** | 4.10 (3.41) | 10.13 (3.44) | **<0.0001** |
| Symptomatology of depression | 8.93 (4.94) | 3.60 (3.53) | **<0.0001** | 2.27 (2.43) | 4.13 (3.82) | 0.07 | 1.90 (2.56) | 6.17 (3.36) | **<0.0001** |

*Note*. = mean; SD= standard deviation. CG: Control Group. EG: Experimental Group. Euroqol-5D-5L: EuroQoL 5-domain for Health-related quality of life. VAS: visual analogue scale. EORTC QLQ-C30: European Organization for Research and Treatment-a cancer –specific measure of Health-related Quality of Life Questionnaire. Global health status and Functional scales (EORTC QLQ-C30): Scores range from 0 to 100, where a higher score represents a higher functional level. Symptom scales and Financial difficulties (EORTC QLQ-C30): Scores range from 0 to 100, where a higher score represents a greater degree of symptoms. HADs: Hospital Anxiety and Depression scale. P-values in bold indicate a significance level of p< 0.05.
