# Supplementary material for: Variables related to health‐related quality of life among breast cancer survivors after participation in an interdisciplinary treatment combining mindfulness and physiotherapy
Source: Cancer Med. 2023 May 11;12(12):13834–45. doi: 10.1002/cam4.6035 (PMC10315809; doi:10.1002/cam4.6035)
Supplement: Supplementary file 3 — Table S2 [file CAM4-12-13834-s003.docx]

**eTable 2. Univariable analysis of Global Health Status (EORTC-QLQ-C30) for breast cancer patients.**

|  | **EORTC QLQ-C30 - Global Health Status** | | | |
| --- | --- | --- | --- | --- |
|  | **6 weeks** | | **3 months** | |
|  | $\bar{x} (sd)$ | p-value | $\bar{x} (sd)$ | p-value |
| *Clinical data* |  |  |  |  |
| Age* | 0.01 | 0.92 | -0.05 | 0.68 |
| Smoker |  | 0.91 |  | 0.98 |
| Non-smoker | 65.97 (22.32) |  | 67.36 (22.60) |  |
| Current smoker | 66.03 (21.64) |  | 69.23 (22.67) |  |
| Former smoker | 60.42 (26.38) |  | 64.58 (29.76) |  |
| Family history of gynaecological cancer |  | 0.99 |  | 0.87 |
| No | 69.20 (19.63) |  | 71.01 (20.39) |  |
| Yes | 67.26 (25.42) |  | 69.64 (27.27) |  |
| Charlson Index categorized |  | 0.96 |  | 0.37 |
| 0 | 68.67 (21.79) |  | 71.67 (22.96) |  |
| 1 | 68.52 (17.57) |  | 65.74 (16.90) |  |
| 2 | - |  | - |  |
| >2 | 75.00 (-) |  | 66.67 (-) |  |
| *Pathological anatomy data* |  |  |  |  |
| Laterality |  | 0.87 |  | 0.37 |
| Left breast | 69.91 (15.72) |  | 73.61 (16.73) |  |
| Right breast | 67.01 (27.20) |  | 66.32 (27.85) |  |
| Cancer stage |  | 0.63 |  | 0.75 |
| 0 | 61.11 (24.96) |  | 68.06 (28.61) |  |
| I | 71.77 (16.34) |  | 72.31 (16.44) |  |
| II | 67.86 (27.51) |  | 70.23 (29.18) |  |
| III | 72.22 (4.81) |  | 66.67 (0) |  |
| IV | - |  | - |  |
| Histological types |  | 0.90 |  | 0.82 |
| Ductal carcinoma in situ | 69.44 (17.16) |  | 77.78 (20.21) |  |
| Invasive ductal carcinoma | 70.20 (18.75) |  | 70.71 (19.11) |  |
| Invasive lobular carcinoma | 59.72 (30.01) |  | 65.28 (32.67) |  |
| Tubular carcinoma | 79.17 (5.89) |  | 66.67 (-) |  |
| Mucinous carcinoma | 83.33 (-) |  | 83.33 (-) |  |
| Medullary | 75.00 (-) |  | 66.67 (-) |  |
| Invasive cribriform | - |  | - |  |
| Invasive papillary | 66.67 (-) |  | 66.67 (-) |  |
| Carcinoma not specified | - |  | - |  |
| Other | 55.56 (50.92) |  | 55.56 (50.92) |  |
| *Neoadjuvant therapy data* |  |  |  |  |
| Pre-intervention treatment type |  | 0.55 |  | 0.79 |
| No | 68.82 (21.22) |  | 70.69 (22.31) |  |
| Yes | 66.67 (11.79) |  | 70.83 (5.89) |  |
| Chemotherapy |  | 0.55 |  | 0.79 |
| No | 68.82 (21.21) |  | 70.69 (22.31) |  |
| Yes | 66.67 (11.79) |  | 70.83 (5.89) |  |
| Radiation |  | 0.31 |  | 0.88 |
| No | 68.93 (21.04) |  | 70.62 (22.12) |  |
| Yes | 58.33 (-) |  | 75.00 (-) |  |
| Hormone therapy |  | na |  | na |
| No | 68.75 (20.90) |  | 70.69 (21.94) |  |
| Yes | - |  | - |  |
| *Quality-of-life questionnaires (baseline)* | |  |  |  |
| Euroqol-5D-5L |  |  |  |  |
| Scale 1 (EQ-5D)* | 0.16 | 0.20 | 0.05 | 0.65 |
| Scale 2 (VAS)* | 0.13 | 0.29 | 0.10 | 0.40 |
| EORTC QLQ-C30 |  |  |  |  |
| Global health status* | 0.59 | **<0.0001** | 0.46 | **0.0002** |
| Functional scales |  |  |  |  |
| Physical functioning* | 0.23 | 0.06 | 0.13 | 0.32 |
| Role functioning* | 0.16 | 0.20 | 0.02 | 0.84 |
| Emotional functioning* | 0.06 | 0.59 | -0.01 | 0.90 |
| Cognitive functioning* | 0.04 | 0.73 | -0.06 | 0.60 |
| Social functioning* | 0.19 | 0.13 | 0.10 | 0.44 |
| Symptom scales |  |  |  |  |
| Fatigue* | -0.11 | 0.39 | -0.01 | 0.93 |
| Nausea and vomiting* | -0.13 | 0.29 | -0.09 | 0.46 |
| Pain* | -0.13 | 0.29 | -0.04 | 0.74 |
| Dyspnoea* | -0.13 | 0.29 | -0.04 | 0.75 |
| Insomnia* | 0.02 | 0.87 | 0.14 | 0.25 |
| Appetite loss* | -0.02 | 0.86 | 0.08 | 0.50 |
| Constipation* | -0.12 | 0.35 | -0.05 | 0.66 |
| Diarrhoea* | -0.10 | 0.42 | -0.05 | 0.65 |
| Financial difficulties* | -0.10 | 0.42 | -0.04 | 0.72 |
| HADs |  |  |  |  |
| Symptomatology of anxiety* | 0.14 | 0.26 | 0.24 | 0.05 |
| Symptomatology of depression* | 0.29 | **0.02** | 0.39 | **0.001** |

*Note*. = mean; SD= standard deviation. *: data are given as Pearson’s correlation. -: not applicable. Euroqol-5D-5L: EuroQoL 5-domain for Health-related quality of life. VAS: visual analogue scale. EORTC QLQ-C30: European Organization for Research and Treatment:-a cancer –specific measure of Health-related Quality of Life Questionnaire. HADs: Hospital Anxiety and Depression scale. P-values in bold indicate a significance level of p< 0.05.
