# Supplementary material for: Variables related to health‐related quality of life among breast cancer survivors after participation in an interdisciplinary treatment combining mindfulness and physiotherapy
Source: Cancer Med. 2023 May 11;12(12):13834–45. doi: 10.1002/cam4.6035 (PMC10315809; doi:10.1002/cam4.6035)
Supplement: Supplementary file 4 — Table S3 [file CAM4-12-13834-s001.docx]

**eTable 3. Satisfaction with treatment at 6 weeks and at 3 months (Experimental group).**

| Satisfaction with treatment | at 6 weeks | at 3 months |
| --- | --- | --- |
|  | **n (%)** | **n (%)** |
| I’m not satisfied | 0 (0) | 0 (0) |
| I’m only somewhat satisfied | 0 (0) | 0 (0) |
| I’m moderately satisfied | 1 (3.33) | 0 (0) |
| I’m very satisfied | 13 (43.33) | 9 (30.00) |
| I am completely satisfied | 16 (53.33) | 21 (70.00) |

Note. Responses to the question “Are you satisfied with the treatment?”. Results are shown as frequencies (percentage).
